# Erratum: Free-standing supramolecular hydrogel objects by reaction-diffusion

**DOI:** 10.1038/ncomms16128

**Published:** 2017-06-30

**Authors:** Matija Lovrak, Wouter E.J. Hendriksen, Chandan Maity, Serhii Mytnyk, Volkert van Steijn, Rienk Eelkema, Jan H. van Esch

Nature Communications 8: Article number:15137 ; DOI: 10.1038/ncomms15317 (2017); Published: 06 05 2017; Updated 06 30 2017

In the original HTML version of this Article, which was published on 5 June 2017, the publication date was incorrectly given as 5 July 2017. This has now been corrected in the HTML; the PDF version of the paper was correct from the time of publication.

