# Supplementary material for: Free-standing supramolecular hydrogel objects by reaction-diffusion
Source: Nat Commun. 2017 Jun 5;8:15317. doi: 10.1038/ncomms15317 (PMC5465320; doi:10.1038/ncomms15317)
Supplement: Supplementary Information — Supplementary Figures, Supplementary Tables, Supplementary Discussion, Supplementary Methods and Supplementary References. [file ncomms15317-s1.pdf]

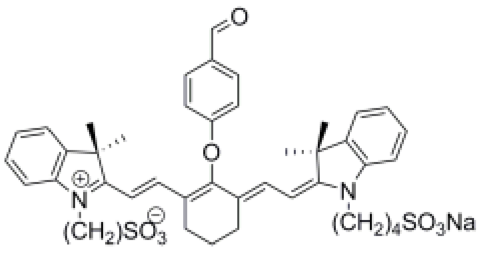

AC

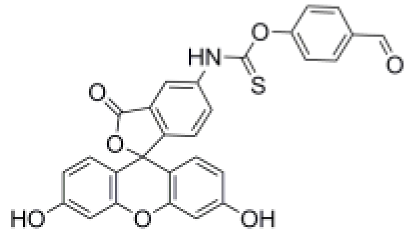

AF

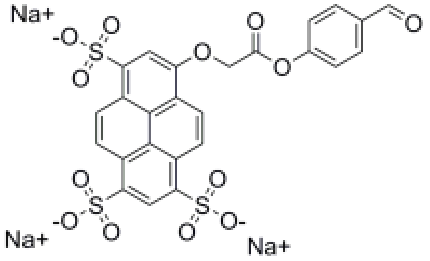

AP

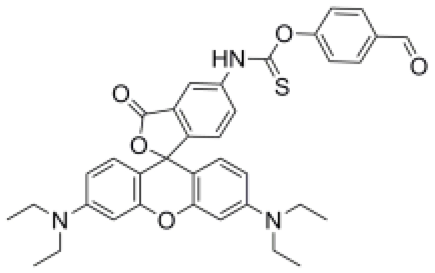

AR

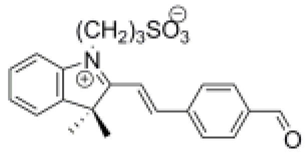

AS

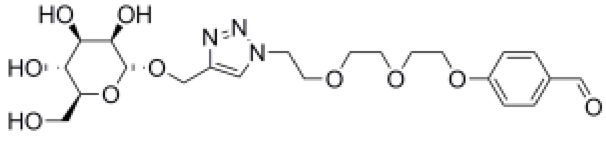

AM

Supplementary Figure 1. Chemical structures of functionalized benzaldehydes. AC is benzaldehyde labeled with cyanine; **AF** is benzaldehyde labeled with fluorescein; **AP** is benzaldehyde labeled with sulfonated pyrene; **AR** is benzaldehyde labeled with rhodamine B; **AS** is benzaldehyde labeled with styryl; **AM** is benzaldehyde labeled with mannose.

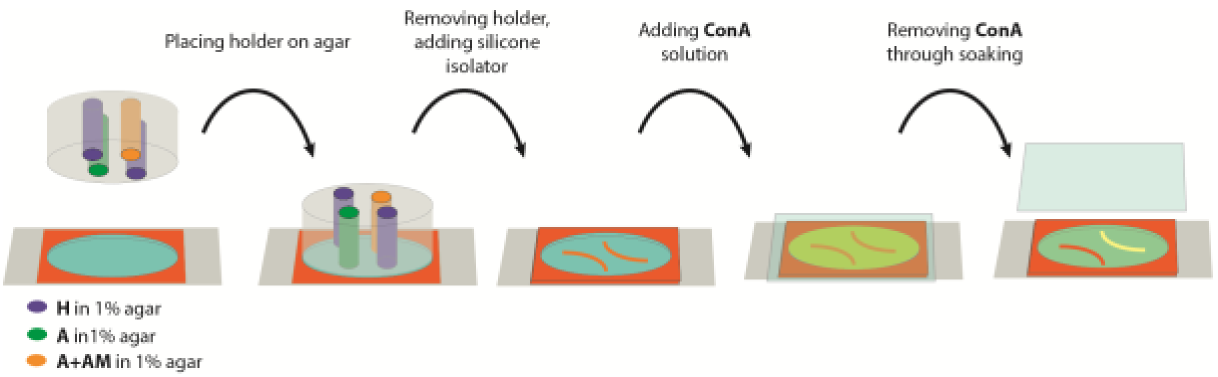

Supplementary Figure 2. Scheme of the process used for the ConA binding setup. A Plexiglass holder was loaded with solutions of hydrazide **H**, aldehyde **A**, and **A** + **AM** in agar. The holder was placed on top of an agar matrix prepared in a well created by putting a Press-to-Seal™ silicone isolator on top of a glass slide and the holder was left standing for 8 hours. Next, the holder was removed, one Press-to-Seal™ silicone isolator was added on top of the previous one and the resulting well was filled with the solution of **ConA** (2 mM, pH = 7). This well was closed with a glass slide and left standing for 12 hours. Afterwards, the solution of **ConA** was removed and replaced with fresh buffer solution to remove non-bound **ConA**. The solution was exchanged every 24 hours for 72 hours in total.

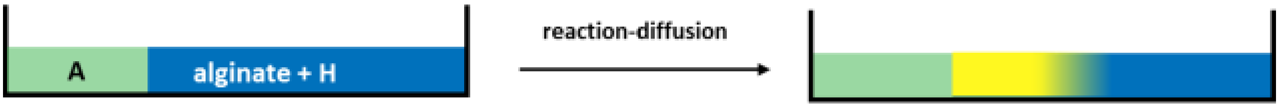

Supplementary Figure 3. The scheme of the formation of hybrid network gels for compression tests. The yellow region represents the alginate/$\mathbf{HA_3}$ hybrid network.

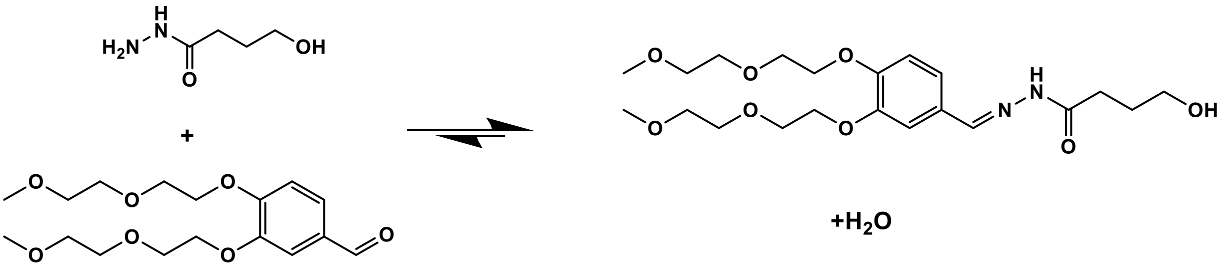

Supplementary Figure 4. One-step reaction for kinetic analysis. A simplified reaction used to determine the pH dependence of reaction rate constant.

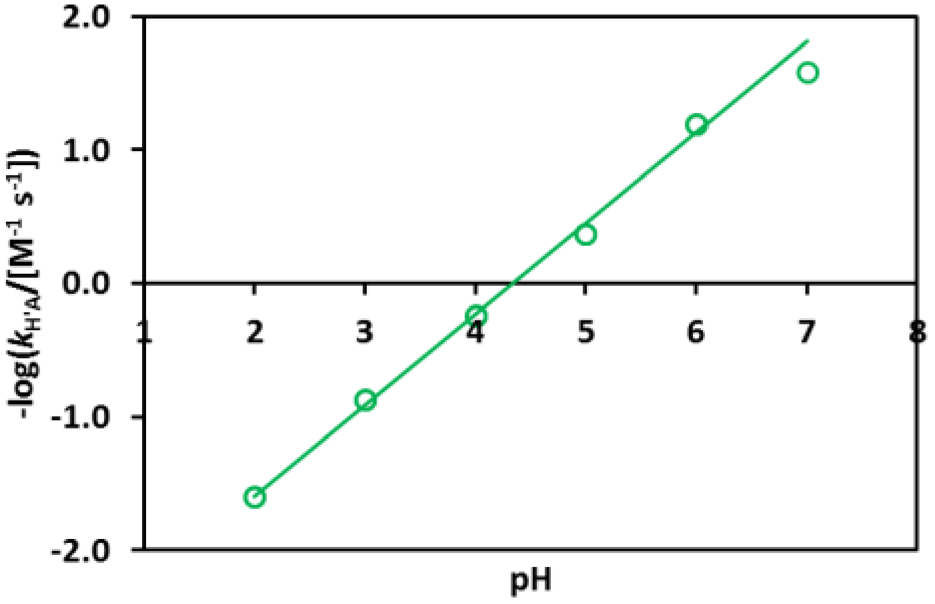

Supplementary Figure 5. Dependence of the rate constant on pH for the one-step reaction.

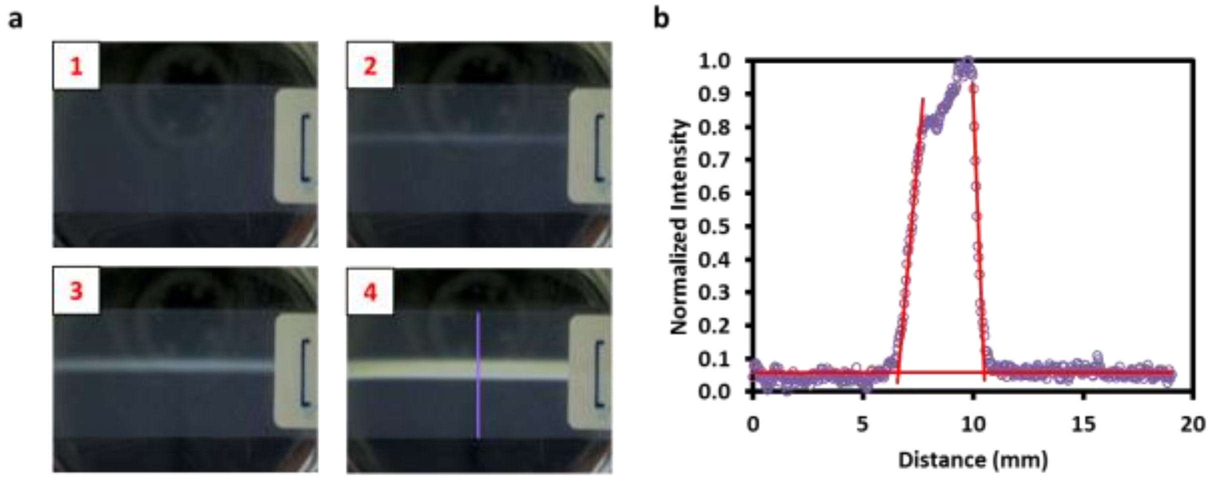

Supplementary Figure 6. Formation of supramolecular hydrogel lines over time at pH = 4.0. a) Photo 1, t = 0 min; photo 2, t = 400 min; photo 3, t = 470 min; photo 4, t = 2000 min **b**) The normalized intensity profile was obtained from the intensity measured along the purple line, shown in photo 4, to determine the temporal development of the width of the 1D pattern as the distance between points where two slopes intersect a base line.

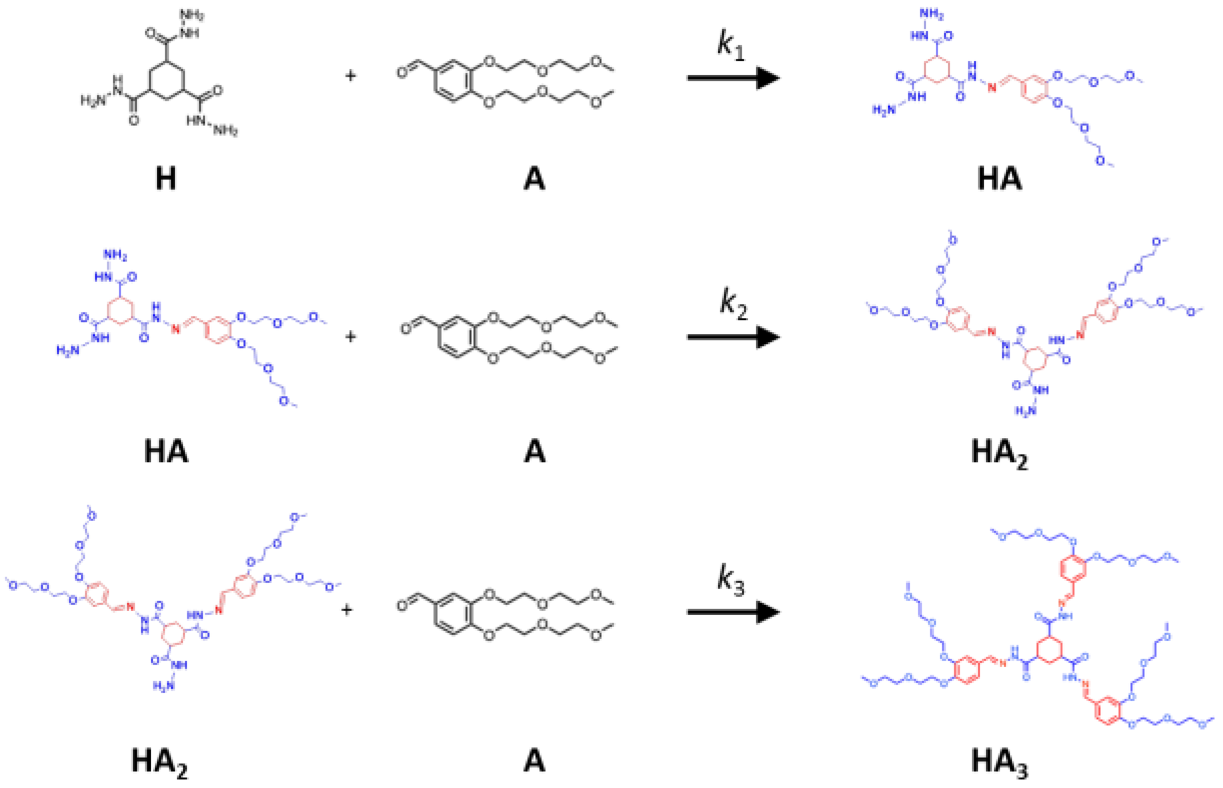

Supplementary Figure 7. Formation of gelator HA$_3$.

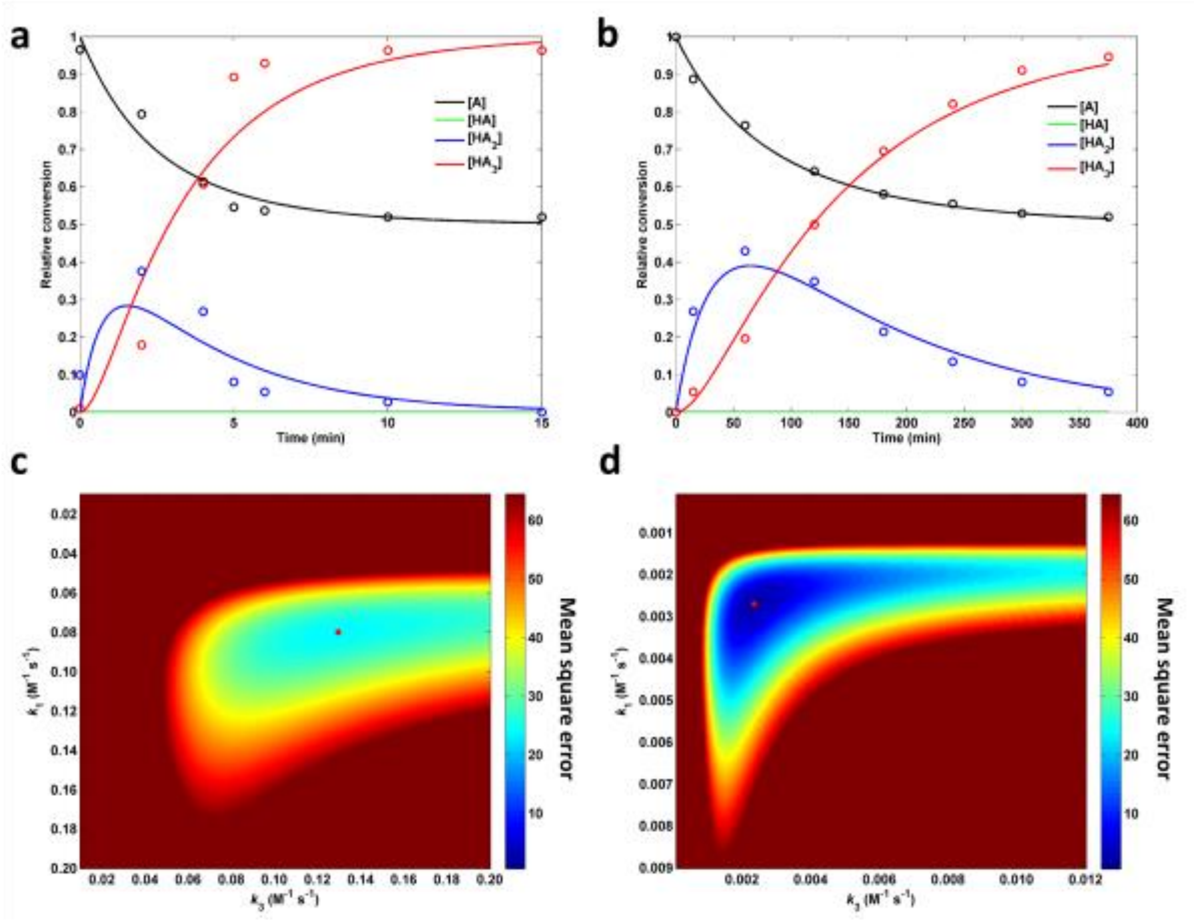

Supplementary Figure 8. The kinetics of gelator formation at pH = 5 (a) and pH = 7 (b). a, b) The experimental data is shown as circles and the lines show the best fit obtained from numerical simulations. The relative conversion for each component was calculated as its concentration normalized by the initial concentration of **H**, except for **A**, which was normalized by its own initial concentration. **c, d)** Plots show mean square error for different combinations of constants k_1 and k_3 at pH = 5 (**c**) and pH = 7 (**d**). The red dot indicates the combination that gave the best fit.

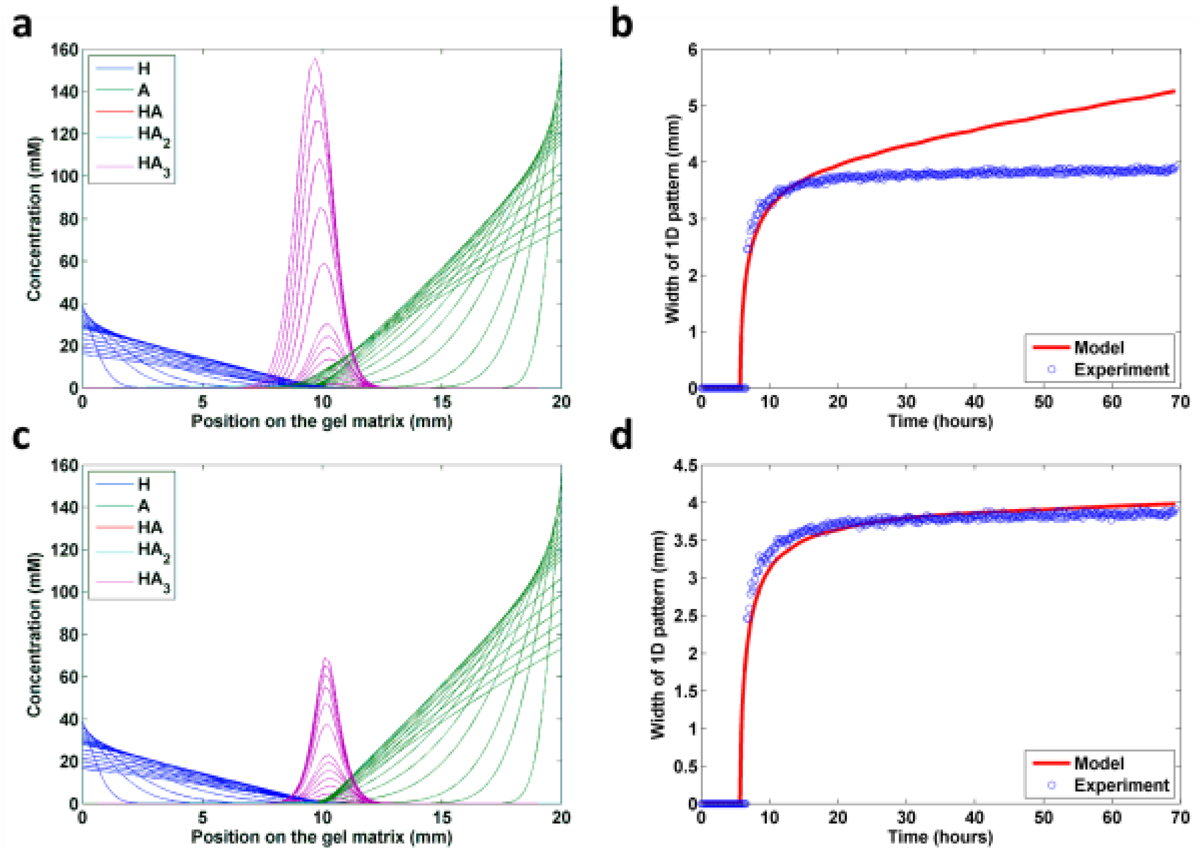

Supplementary Figure 9. Time-dependent structure formation in the RD model at pH = 4.0. a) The concentration profiles of all species in space and time for the model without the decrease of diffusion coefficient of all species depending on the concentration of **HA_3**. **b)** The width of 1D pattern for the model without the decrease of diffusion coefficient of all species depending on the concentration of **HA_3**. **c)** The concentration profiles of all species in space and time for the model with the decrease of diffusion coefficient of all species depending on the concentration of **HA_3**. **d)** The width of 1D pattern for the model with the decrease of diffusion coefficient of all species depending on the concentration of **HA_3**. Different lines in **a** and **c** represent different time points with the first 10 lines being separated by 1.7 hours and the following lines being separated by 8.3 hours. The width of the 1D pattern from the model was extracted from the concentration profile of **HA_3** (as a horizontal distance between two points of the profile at 1 mM).

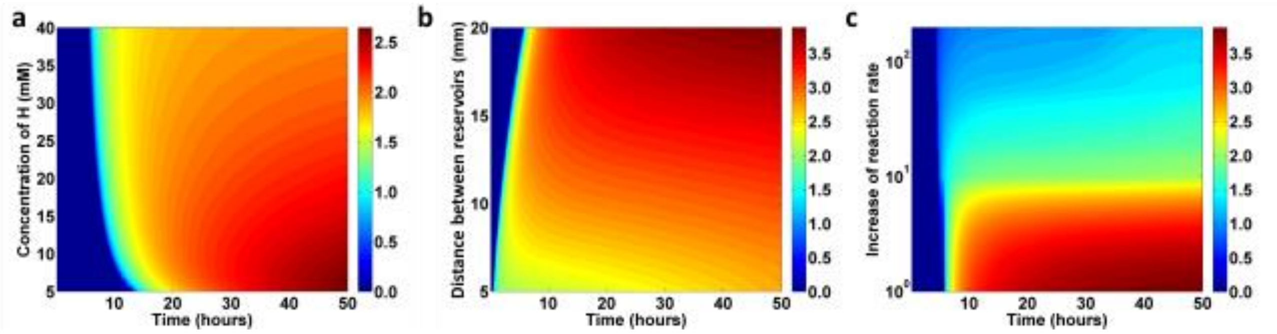

Supplementary Figure 10. Predictions from the model. a) Effect of initial concentrations of **H** on the width of the 1D pattern. **b)** Effect of distance between reservoirs with **H** and **A** on the width of 1D pattern. **c)** Effect of rate constant on the width of 1D pattern. The increase in reaction rate designates how many times the reaction rate constants k_1, k_2, and k_3 are bigger than their values at pH = 4.0. The color bars represent the width of 1D pattern in mm. It should be noted that dark blue areas represent the time points when pattern has not started forming yet, i.e. the **HA₃** concentration is below 1 mM.

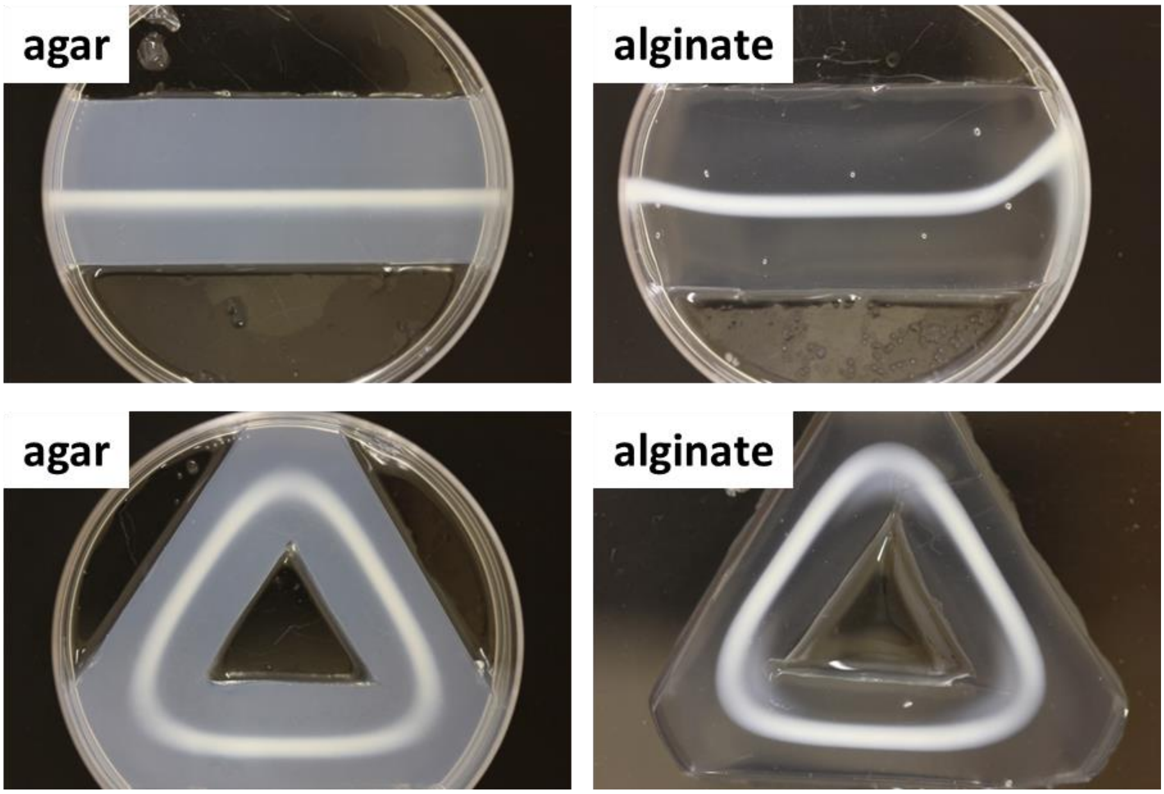

Supplementary Figure 11. Comparison between agar and alginate. All experiments were conducted using the same basic conditions: 40 mM **H** and 160 mM **A** at pH = 4.5 in a 5 cm Petri dish. The only difference was either the use of agar (1 %, left) or calcium alginate (1 %, right).

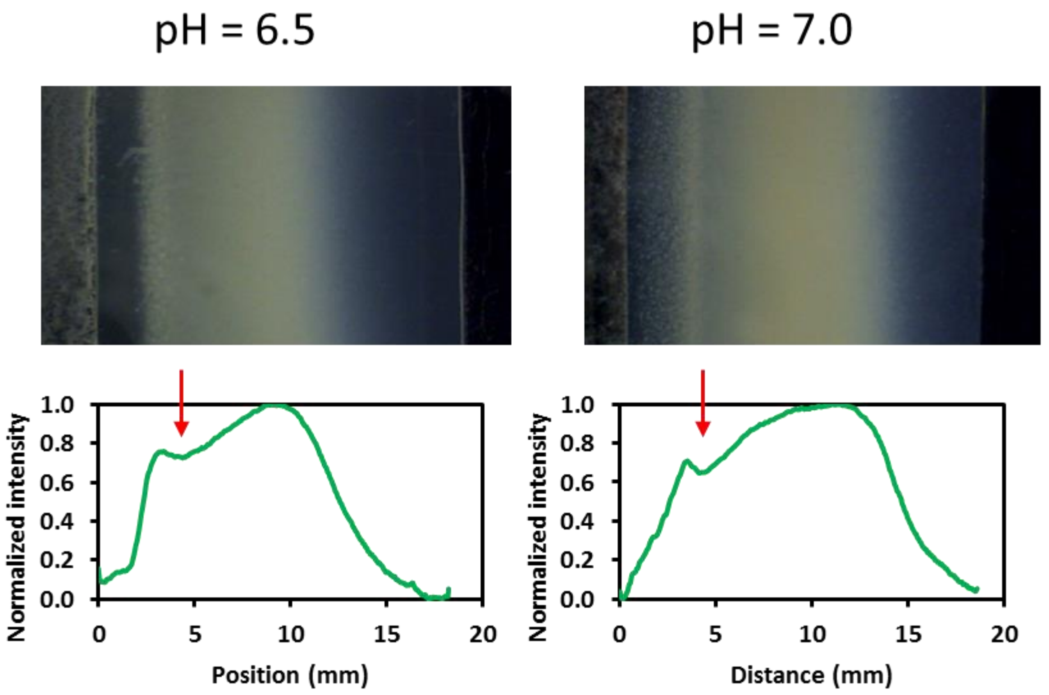

Supplementary Figure 12. Band formation at high pHs. All experiments were conducted using the same basic conditions: 40 mM **H** and 160 mM **A** at *d* = 2 cm. Red arrow indicates the band with less **HA_3**.

Supplementary Table 1. Dependence of rate constant on pH for the one-step reaction as determined from UV-VIS measurements.

pH	$k_{H'A}$ [M^{-1} s^{-1}]
2.0	40
3.0	7.4
4.0	1.7
5.0	4.3×10^{-1}
6.0	6.4×10^{-2}
7.0	2.6×10^{-2}

Supplementary Table 2. Rate constants of HA_3 formation. The rate constants were obtained from the kinetic model by determining the smallest error between the experimental data and the data from the model. The rate constants of the one-step reaction are given for comparison.

pH	$k_{H'A}$ [M^{-1} s^{-1}]	k_1 [M^{-1} s^{-1}]	k_3 [M^{-1} s^{-1}]
5.0	4.3×10^{-1}	0.8×10^{-1}	1.3×10^{-1}
7.0	2.6×10^{-2}	2.7×10^{-3}	2.4×10^{-3}

Supplementary Table 3. Parameters used in the RD model. The column "Default value" indicates values that were used throughout the model. The column "If changed" indicates other values that were used in certain instances. Only one parameter was changed at a time while keeping other parameters at their default values. Column "Shown" indicates which figures show the result of changing parameters.

Parameter	Default value	If changed	Shown
$D_{\mathbf{H}}$	5.40×10^{-6} cm^2 s^{-1}		
$D_{\mathbf{A}}$	4.50×10^{-6} cm^2 s^{-1}		
$D_{\mathbf{HA}}$	2.76×10^{-6} cm^2 s^{-1}		
$D_{\mathbf{HA2}}$	1.95×10^{-6} cm^2 s^{-1}		
$D_{\mathbf{HA3}}$	10^{-11} cm^2 s^{-1}		
pH (determines rate constants)	4	2 – 7	Supplementary Figure 12c
Distance between reservoirs, *L*	20 mm	5 – 20 mm	Supplementary Figure 12b
$c_{\mathbf{H}}$	40 mM	5 – 40 mM	Supplementary Figure 12a
$c_{\mathbf{A}}$	160 mM	$4 \times c_{\mathbf{H}}$	Supplementary Figure 12a

Supplementary Methods

Synthesis of AP

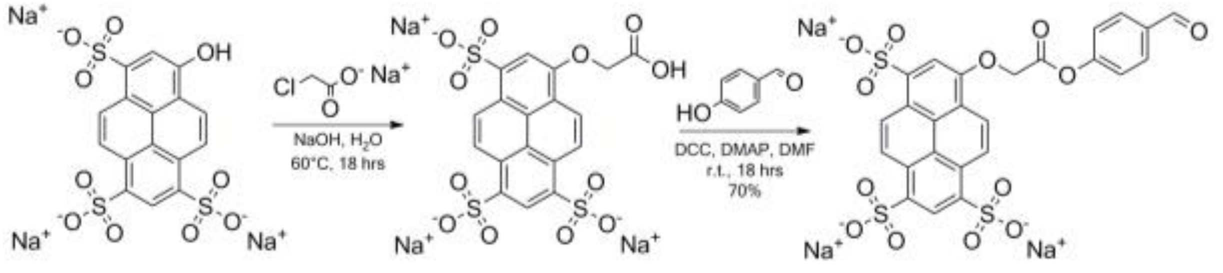

1 2 AP

AP was prepared from **1** by carboxymethylation followed by esterification with 4-hydroxybenzaldehyde. Both steps were adapted from reported methods.[1,2]

8-Hydroxypyrenetrisulfonate trisodium salt **1** (1 g), sodium hydroxide (92 mg, 1.2 eq.), and sodium chloroacetate (335 mg, 1.2 eq.) were dissolved in demineralized water (25 ml). The resulting mixture was subsequently heated to 60 °C, magnetically stirred for 18 hours, cooled down to room temperature, acidified with hydrochloric acid to pH = 3.0, and dried in vacuum. Dry residue was repeatedly washed with boiling ethanol and dried overnight at 60°C in vacuum to afford 685 mg of **2**. ^1H NMR (400 MHz, D_2O) δ, ppm: 9.13 (s, 1H), 9.07 (d, 1H), 8.93 (q, 2H), 8.68 (d, 1H), 8.26 (s, 1H)

Next, DMAP (120 mg, 1 eq.) and 4-hydroxybenzaldehyde (412 mg, 3 eq.) were added to the solution of **2** (680 mg) in anhydrous DMF (20 ml). DCC (255 mg, 1.1 eq.) was then added and the mixture was stirred overnight at 25°C. DMF was removed in vacuum. Solid residue was repeatedly washed with boiling ethanol to remove impurities, and dried overnight at 60°C in vacuum to afford 600 mg of **AP**. ^1H NMR (400 MHz, D_2O) δ, ppm: 9.31 (s, 1H), 9.10 (s, 1H), 9.02 (d, 1H), 8.83 (q, 2H), 8.63 (d, 1H), 8.16 (s, 1H), 7.34 (d, 2H), 6.53 (d, 2H); ^{13}C NMR (100 MHz, D_2O) δ, ppm: 197.12, 196.79, 166.72, 158.81, 142.09, 136.31, 136.19, 135.36, 133.57, 132.61, 129.99, 129.04, 128.95, 127.72, 127.29, 127.06, 125.96, 124.12, 123.68, 121.19, 118.76, 118.65, 53.33; ESI-MS (neg. mode): 257 (hydrolyzed AP+2H^+); 558 (hydrolyzed AP+2Na^+). We could not observe the molecular ion either by ESI or MALDI-ToF MS.

Labeling of alginate with BODIPY® TR Cadaverine (5-(((4-(4,4-Difluoro-5-(2-Thienyl)-4-Bora-3a,4a-Diaza-s-Indacene-3-yl)phenoxy)acetyl)amino)pentylamine, Hydrochloride)

Alginate-BODIPY TR conjugate was prepared using the following procedure. 1-Ethyl-3-(3-dimethylaminopropyl)carbodiimide hydrochloride (EDC, 3.67 mg) and *N*-hydroxysuccinimide (NHS, 2.57 mg) were added to a solution of sodium alginate (104.1 mg) in water (10 mL), followed by the addition of a solution of BODIPY® TR Cadaverine (1.16 mg) in DMSO (100 μL). The resulting mixture was stirred overnight at 25°C, transferred to a dialysis tube (Spectra/Por®, MWCO = 6500 Da), dialyzed against 5 × 1 L of demineralized water over 3 days, and freeze-dried. The resulting powder was stored at -20 °C.

RD-SA by drop deposition (Figure 2g)

To form letters by RD-SA, we used the following procedure. A plastic Petri dish (8.5 cm in diameter) was filled with an agar solution and left to gelate. This layer acts a the diffusion and reaction medium. After cooling, solutions containing **H** (40 mM) and **A** (160 mM) in agar were deposited as droplets on top of the agar layer. The droplets (50 μL) were pipetted at opposite sides of the outline of the letters, along their contour (see the cartoon in Figure 5g), forming the "ASM" abbreviation. Careful deposition prevented direct contact between droplets, which gelate in less than a minute. The sample was left standing for ~5 hours in a closed humidified environment to allow the diffusion of **H** and **A**.

Chemically differentiated grid (Figure 5a)

To make the chemically differentiated grid, we followed the procedure described in section 3.2, except for filling some of the reservoirs with solutions of **A** containing different benzaldehyde-functionalized dyes (**AC**, **AF** or **AS**), according to the configuration shown in Supplementary Figure 3a. The concentration of **A** was 160 mM if only **A** was used and 144 mM if mixed with the dye. The concentration of dye was 10 mM.

Chemical differentiation by fluorescent labeling of RD-SA patterns (Figure 5b)

In this experiment, we used a cylindrical Plexiglass holder to deliver compounds into the gel matrix. The holder was 2.5 cm in diameter and 2 cm in height with four through holes symmetrically staggered around the center. The holes were 2 mm in diameter and their centers 5 mm apart. To chemically label the formed RD-SA patterns, we prepared solutions of **A** (160 mM) **+ AF** (40 µM) and **A** (160 mM) **+ AR** (40 µM) in agar. These two solutions were injected in two holes, while the other holes were filled with a solution containing **H** (40 mM), see Supplementary Figure 4 and Figure 3b. The solutions were left to gelate for 20 minutes after which the holder was placed on a flat agar matrix and left standing for 8 hours in a closed humidified environment. The resulting patterns were analyzed by confocal microscopy, using the following settings: **AF** (λ_{ex} = 488 nm, λ_{em} = 500-550 nm), **AR** (λ_{ex} = 548 nm, λ_{em} = 580-700 nm).

Chemical gradient (Figure 5c)

An agar matrix loaded with **H** (40 mM) was prepared in a Petri dish. A reservoir for the solution of **A** was made by manually cutting out a circular segment of agar (the central angle defining the circular segment was 110°), see Supplementary Figure 3c. The solution of **A** (200 mM) **+ AF** (8 mM) was placed in the reservoir and the setup was left standing for 2 weeks. The solutions and agar matrix were prepared using phosphate buffer of pH = 6.5.

Chemical labeling for enzyme binding to RD-SA patterns (Figure 5e)

To make RD-SA patterns capable of binding ConcavalinA (**ConA**), solutions of **A** (160 mM) **+ AR** (40 µM) and **A** (160 mM) **+ AM** (1.6 mM) **+ AR** (40 µM) in agar were prepared. These solutions were pipetted in two separate holes in a Plexiglass holder. The other two holes were filled with a solution containing **H** (40 mM) according to the configuration shown in Supplementary Figure 4 and Supplementary Figure 3d, and the solutions were left to gelate. The experiment was then performed according to the procedure in Supplementary Figure 4. In short: the holder was placed on top of the agar gel prepared in the well formed by putting a Press-to-Seal™ silicone isolator on a glass slide and left to diffuse in a closed humidified environment for 8 hours. The time was kept intentionally short to prevent the formation of a dense supramolecular network, which would not allow the **ConA** to penetrate. Subsequently, the holder was removed and another Press-to-Seal™ silicone isolator was placed on top and the resulting well was filled with a buffered solution of **ConA** (2 mM, pH = 7). This well was closed with a glass slide to prevent evaporation and left to stand in a closed humidified environment for 12 hours. After this period, the sample was analyzed using fluorescence microscopy. To remove the non-bound **ConA**, the top glass slide was removed, the **ConA** solution was removed, and the sample was immersed into buffer (at least several mL) and left to stand for 72 hours, while buffer being refreshed every 24 hours. The final sample was again imaged by fluorescence microscopy. Images were analyzed with ImageJ to extract the fluorescence intensity profiles.

Formation of hybrid network materials for compression tests (Figure 3a/b)

Alginate gels containing **H** were prepared similarly to pure alginate gels, but now dispersing $CaCO_3$ powder in an aqueous solution of **H** instead of in water. 10 mL of this solution was added to a Petri dish and left to gelate. Then, we manually cut out a circular segment of agar (the central angle defining the circular segment was 110°) to make a reservoir (Supplementary Figure 5). The solution of **A** was placed in the reservoir and the reaction was left running for at least three weeks (until the patterned region was more than 1 cm wide). Cylinders of 8 mm in diameter were punched out from the alginate/$\mathbf{HA_3}$ hybrid network region using a circular puncher and subjected to compression tests.

To prepare gels with varying initial concentration of **H** in alginate (10, 20, 30, 35 and 40 mM **H**), the following conditions were kept constant: w(alginate) = 1.5 %, $c(CaCO_3)$ = 12.5 mM, c(**GDL**) = 40 mM, c(**A**) = 400 mM. The final pH of the gel depends on the ratio of concentrations of $CaCO_3$ and **GDL**. The current conditions gave a pH of 4.5. It should be noted that when the initial concentration of **H** in alginate was 30 mM, the final concentration of $\mathbf{HA_3}$ in hybrid network gel was around 70 mM. Since the solubility of **H** in water is 40 mM we could not measure the yield stress of pure 70 mM $\mathbf{HA_3}$. Nevertheless, we do not expect that it would be significantly higher than the yield stress of 30 mM $\mathbf{HA_3}$, especially taking into account that the pure 30 mM $\mathbf{HA_3}$ was physically so weak (as confirmed by the yield stress of only 0.7 kPa) that it was challenging to transfer it from a Petri dish to the instrument for measurement. Therefore, we have not attempted to measure pure 10 mM and 20 mM $\mathbf{HA_3}$ to get a trend of increase of mechanical properties versus the concentration of $\mathbf{HA_3}$**.** Because of noted and confirmed low mechanical performance of 30 mM $\mathbf{HA_3}$ we assumed that the increase of mechanical properties of $\mathbf{HA_3}$ versus the concentration of $\mathbf{HA_3}$ cannot be significant compared to the effect of the formation of hybrid network.

To prepare optimally crosslinked gels with varying mass fraction of alginate (1, 2 and 3 %), the following conditions were kept constant: c_{initial}(**H** in alginate) = 30 mM, c(A) = 400 mM, pH = 4.5. The optimal crosslinking was controlled by adjusting the amount of $CaCO_3$, and the final pH (4.5) was controlled by adjusting the amount of **GDL**.

The kinetics of hydrazone formation, measured using one-step reaction

We explored how the reaction constant of hydrazone formation varies with pH. Since the formation of $\mathbf{HA_3}$ is a three-step reaction, it would be complicated to investigate its dependence on pH. Therefore, we used a one-step reaction of monohydrazide **H'** with aldehyde **A** giving monohydrazone **H'A** (Supplementary Figure 6). This reaction can be easily followed using UV-VIS spectroscopy. Briefly, the absorbance of a well-mixed reaction mixture containing **H'** (60 µM) and **A** (60 µM) was measured at 308 nm until no significant change in absorbance was observed. The evolution of the concentration of **H'A** was obtained from the absorbance curve using a separately constructed calibration curve. The initial rate of **H'A** formation was determined from the linear least-square fit of the initial increase of **H'A** concentration, and used in combination with the initial concentrations of **H'** and **A** to calculate the rate constant under the assumption that the kinetics of the reaction is first order in both reactants, i.e. $\mathrm{d}c_{\mathbf{H'A}}/\mathrm{d}t = k_{\mathbf{H'A}}c_{\mathbf{H'}}c_{\mathbf{A}}$. The resulting rate constants for different pHs are summarized in Supplementary Table 1 and Supplementary Figure 7, and agree well with reported values for a similar hydrazone formation reactions.[3]

Dynamics of 1D pattern formation

To study the dynamics of 1D pattern formation, we performed a set of experiments using the basic configuration shown in Figure 2a in which the width of the 1D pattern increases over time. A plastic Petri dish was filled with agar at the desired pH and left to gelate. A 2 cm wide agar strip was made with two parallel cuts, at equal distance from the center. The outer agar segments were removed, creating two reservoirs. Before injecting buffer solutions of **H** and **A** in these reservoirs, the Petri dish was placed on a flat Plexiglass plate above a digital microscope camera. The recording started at the moment that the two solutions were injected. Images were taken every 10 minutes, over the course of 20 to 120 hours depending on experimental conditions. They were analyzed using ImageJ, extracting the intensity profile along a line between the two reservoirs (Supplementary Figure 8a, purple line in photo 4). This was done for multiple lines in the same image and the resulting profiles were averaged to reduce noise. The resulting intensity profile was then normalized by subtracting the minimum intensity value from the raw intensity profile and subsequently dividing the resulting intensities with respect to the peak intensity. The width of the 1D pattern was determined as the distance between points where two slopes intersect a base line (Supplementary Figure 8b). This baseline was determined as the average value of the first ten points of the intensity profile. This was done for all images in a time lapse, yielding the evolution of the width of the formed 1D pattern.

Kinetic model for HA_3 formation

The formation of gelator $\mathbf{HA_3}$ proceeds in three consecutive steps as shown in Supplementary Figure 9. Assuming that the rate constants of the three steps k_1, k_2, and k_3 are independent and that they are first order in reactant concentration, the concentration of species in a well-mixed system without diffusion limitations changes in time according to:

$$\frac{d[\mathrm{H}]}{dt} = -k_1[\mathrm{H}][\mathrm{A}] \tag{1}$$

$$\frac{d[\mathrm{A}]}{dt} = -k_1[\mathrm{H}][\mathrm{A}] - k_2[\mathrm{HA}][\mathrm{A}] - k_3[\mathrm{HA_2}][\mathrm{A}] \tag{2}$$

$$\frac{d[\mathrm{HA}]}{dt} = k_1[\mathrm{H}][\mathrm{A}] - k_2[\mathrm{HA}][\mathrm{A}] \tag{3}$$

$$\frac{d[\mathrm{HA_2}]}{dt} = k_2[\mathrm{HA}][\mathrm{A}] - k_3[\mathrm{HA_2}][\mathrm{A}] \tag{4}$$

$$\frac{d[\mathrm{HA_3}]}{dt} = k_3[\mathrm{HA_2}][\mathrm{A}]. \tag{5}$$

To validate this model and find the values of the rate constants, we performed a set of experiments in a system where diffusion plays no role. Briefly, we mixed solutions of **H** (20 mM) and **A** (120 mM) and measured the concentrations using HPLC to find the temporal concentration changes of **A**, **HA**, $\mathbf{HA_2}$ and $\mathbf{HA_3}$ (see reference 2 for complete experimental procedure details). We compared the experimental data with the concentration data obtained by solving the model equations using a MATLAB code using the same initial concentrations for **A** and **H** as in the experiment. We solved the set of equations for a wide range of reaction constants k_1 and k_3, while k_2 was set to a value for which no significant amounts of the intermediate **HA** were observed (k_2 was at least 1000 × higher than k_1, k_3), based on experimental observations. For each combination of rate constants k_1 and k_3, the goodness of fit between experimental and numerical data was determined as the sum of mean square errors in the concentrations of **A**, $\mathbf{HA_2}$ and $\mathbf{HA_3}$ (Supplementary Figure 10). The rate constants k_1 and k_3 that gave the smallest error are summarized in Supplementary Table 2 for two values of the pH, and are within one order of magnitude as the rate constant for the one-step reaction $k_{\mathbf{H'A}}$. The corresponding concentration data is in good agreement with the experimental data, suggesting the validity of the model assumptions.

Based on the linear dependence between -log $k_{\mathbf{H'A}}$ and pH found for the one-step reaction, we assume that -logk_1, -logk_2 and –logk_3 also linearly depend on pH as the $\mathbf{HA_3}$ reaction involves the same type of bond, but three times instead of once. Using the data in Supplementary Table 2 for k_1 and k_3, we find

$$k_1 = 10^{-(0.74\text{pH}+0.42)} \tag{6}$$

$$k_3 = 10^{-(0.87\text{pH}-0.45)}. \tag{7}$$

Reaction-diffusion model for 1D pattern formation

Having determined the rate constants and their dependency on pH, we developed a reaction-diffusion model to describe the formation of a 1D pattern in the basic experiment shown in Figure 2a. We hereby used the experimentally measured 1D pattern widths (Supplementary Section 6) to validated the model and to find the values of the diffusion coefficients. The reaction-diffusion model is based on Fickian diffusion and described by the following set of partial differential equations.

$$\frac{\partial c_{\text{H}}(x,t)}{\partial t} = D_{\text{H}} \frac{\partial^2 c_{\text{H}}(x,t)}{\partial x^2} - k_1 c_{\text{H}}(x,t) c_{\text{A}}(x,t) \tag{8}$$

$$\frac{\partial c_{\text{A}}(x,t)}{\partial t} = D_{\text{A}} \frac{\partial^2 c_{\text{A}}(x,t)}{\partial x^2} - k_1 c_{\text{H}}(x,t) c_{\text{A}}(x,t) - k_2 c_{\text{HA}}(x,t) c_{\text{A}}(x,t) - k_3 c_{\text{HA}_2}(x,t) c_{\text{A}}(x,t) \tag{9}$$

$$\frac{\partial c_{\text{HA}}(x,t)}{\partial t} = D_{\text{HA}} \frac{\partial^2 c_{\text{HA}}(x,t)}{\partial x^2} + k c_{\text{H}}(x,t) c_{\text{A}}(x,t) - k c_{\text{HA}}(x,t) c_{\text{A}}(x,t) \tag{10}$$

$$\frac{\partial c_{\text{HA}_2}(x,t)}{\partial t} = D_{\text{HA}_2} \frac{\partial^2 c_{\text{HA}_2}(x,t)}{\partial x^2} + k c_{\text{HA}}(x,t) c_{\text{A}}(x,t) - k c_{\text{HA}_2}(x,t) c_{\text{A}}(x,t) \tag{11}$$

$$\frac{\partial c_{\text{HA}_3}(x,t)}{\partial t} = D_{\text{HA}_3} \frac{\partial^2 c_{\text{HA}_3}(x,t)}{\partial x^2} + k c_{\text{HA}_2}(x,t) c_{\text{A}}(x,t). \tag{12}$$

The gel was considered as a one-dimensional (1D) domain, with edges at x = 0 and x = L in contact with the **H** and **A** reservoir, respectively. Initially, concentrations of **HA**, **HA$_2$**, and **HA$_3$** are zero everywhere. Similar is valid for **A** and **H**, except at the edges of the domain where we used (in the notation $c(x, t)$): $c_{\mathbf{H}}(0, 0)$ = 40 mM and $c_{\mathbf{A}}(L, 0)$ = 160 mM. The edges of the domain were considered perfect sinks for the products ($c_{\mathbf{HA3}}(0, t)$ = $c_{\mathbf{HA3}}(\text{L}, t)$ = 0 mM, alike for **HA** and **HA$_2$**), while the concentrations of **A** and **H** at the edges were updated each time step, accounting for depletion of reactants in the reservoirs. The set of equations, together with the initial and boundary conditions, was solved numerically using a MATLAB code. The output gives the spatiotemporal concentration profiles of all species, which can be used to calculate the evolution of the width of the 1D pattern. We determined the width from the time-dependent concentration profile of **HA$_3$** as the horizontal distance between the points where the concentration of **HA$_3$** equals 1 mM. To determine the diffusion coefficients of all species (except for **HA$_3$**, which is virtually 0), we performed a set of simulations in which we varied their values over a wide range. From this set, we determined the best fit to the experimental data of the width of the 1D pattern formed at pH = 4.0 to find their values

(Supplementary Table 3). Comparing the resulting pattern predicted by the model with that observed in experiments at pH=4, we observed that the simulated 1D pattern keeps increasing in width, while the experimental pattern reaches a plateau, as shown in Supplementary Figure 11a. By taking samples from the reservoirs, we excluded depletion of reactants as the underlying reason for the plateau. We hypothesized that the difference in dynamics is due to the gelation of **HA₃**, which makes it harder for the reactants and intermediates to diffuse through the domain. We took into account the local dependence of diffusion coefficients on the local **HA₃** concentration using a stretched exponential function of the form

$$D_g = D_0 \exp\left(-a\varphi^{\upsilon}\right) \tag{13}$$

with φ the volume fraction of **HA₃** in the agar gel matrix modeled as[4]

$$\varphi = \frac{w_{\mathrm{HA_3\ in\ 1\%\ agar}}}{1.025} = \frac{c_{\mathrm{HA_3}} M_{\mathrm{HA_3}} V_{\mathrm{HA_3}}}{1.025 \times (c_{\mathrm{HA_3}} M_{\mathrm{HA_3}} V_{\mathrm{HA_3}} + m_{\mathrm{1\%\ agar}})} . \tag{14}$$

We assumed negligible volume changes upon gelation of solutions, i.e. a certain volume of solution gives the same volume of gel. For simplicity, we set the parameter υ = 1 and used the values in Supplementary Table 3 for D_0. Using the approach similar to the one described before for the determination of D_0, we analyzed the ranges of constant *a* for reactants and intermediates to determine the values that gave the best fit with experimental data. The best fit was found for *a* = 30 for **H**, and *a* = 34 for **A**. We noted that the wide range of values around *a* = 30 for **HA** and **HA₂** does not show any influence on the outcome of the model so we set it to *a* = 34, the same as for **A**. With all parameters optimized at pH = 4.0, we obtained good agreement between model and experiment, as shown in Supplementary Figure 11b, showing the importance of taking into account the dependence of the diffusion coefficients on local gelation.

Finally, we used the optimized model to investigate how changes of different experimentally controllable parameters influence the width of 1D pattern. The results of these simulations are shown in Supplementary Figure 12.

Supplementary Discussion

When comparing the width of the 1D pattern for different concentrations of **H** at a fixed ratio of **H** and **A** concentrations, low concentrations yield wider structures than high concentrations when compared at the same point in time (except for very short times (<20 hours)). This is understood, as higher concentrations yield tougher **HA_3** gels such that it takes longer for reactants and intermediates to diffuse through. This same effect in seen for a decreasing distance between the reservoirs, which yields tougher gels earlier on, slowing down the formation of the 1D pattern. The effect of pH was studied by varying the reaction rate constants in the model. We hereby used the experiment at pH = 4.0 as a benchmark, such that a 200-fold increase in reaction rates corresponds to a decrease in pH to 0.9. Although the pH in our experiments cannot be decreased below 3.3 (as gelation does not occur for the agar and alginate/$CaCO_3$/**GDL** systems) resulting in about 2 mm wide lines, the model does enable exploring how the width of the 1D pattern further decreases with pH. As can be seen in Supplementary Figure 12c, pH strongly influenced the line width, reaching 1.0 mm at pH = 0.9. In summary, the model demonstrates that the smallest patterns could be made using the highest concentrations of reactants possible, keeping the smallest feasible distances between reservoirs, while taking care that reaction rate constants are the highest possible (controlled by pH). Additionally, by knowing only the reaction rates and the diffusion coefficients of participating species, the model can be used to estimate minimum and maximum sizes of produced patterns and is not limited to the reaction used in this research. In our case, the minimum size estimated using the model was in the order of 2 mm, and maximum size was in the order of 15 mm, thus spanning over approximately one order of magnitude.

Supplementary References

1 Jimenez, F. *et al.* Aryloxyacetic esters structurally related to alpha-asarone as potential antifungal agents. *Med. Chem. Res.* **19**, 33-57 (2010).

2 Neises, B. & Steglich, W. 4-Dialkylaminopyridines as acylation catalysts .5. Simple method for esterification of carboxylic-acids. *Angew. Chem. Int. Ed.* **17**, 522-524 (1978).

3 Dirksen, A., Dirksen, S., Hackeng, T. M. & Dawson, P. E. Nucleophilic catalysis of hydrazone formation and transimination: Implications for dynamic covalent chemistry. *J. Am. Chem. Soc.* **128**, 15602-15603 (2006).

4 Johnson, E. M., Berk, D. A., Jain, R. K. & Deen, W. M. Diffusion and partitioning of proteins in charged agarose gels. *Biophys. J.* **68**, 1561-1568 (1995).
